# Innovative Nanomaterials for Biomedical Applications

**DOI:** 10.3390/nano12091561

**Published:** 2022-05-05

**Authors:** Michele Bianchi, Gianluca Carnevale

**Affiliations:** 1 Center for Translational Neurophysiology of Speech and Communication, Fondazione Istituto Italiano di Tecnologia, Via Fossato di Mortara 17, 44121 Ferrara, Italy; 2 Department of Surgery, Medicine, Dentistry and Morphological Sciences with Interest in Transplant, University of Modena and Reggio Emilia, Via del Pozzo, 71, 41124 Modena, Italy; gianluca.carnevale@unimore.it

Research focusing on innovative nanomaterials for applications in biomedicine and bioengineering has steadily gained attention over the last 20 years. This is due to the unique physical and chemical characteristics that can be provided by nanomaterials compared to their bulk counterparts, including augmented surface reactivity; improved mechanical, electrical, magnetic, optical and thermal properties; and their enhanced bioactivity, drug loading capacity or permeability through biological barriers. Advanced nano-biomaterials with tailored surface topography and chemistry can be designed to create environmental conditions favorable for proper protein and consequently for accelerated cell adhesion, proliferation and differentiation [1,2]. In this Special Issue entitled “Nanostructured Materials for Biomedicine and Bioengineering”, we have collected 11 articles (7 original research articles and 4 review articles) that highlight the growing role of nanostructured materials in biomedicine, focusing on their characteristics at the micro- and nano-scale to better characterize the efficiency and functionality of new materials and devices.

Nanostructured modifications of metal scaffolds represent a promising approach to accelerate implant osseointegration through the increasing in endothelial commitment of mesenchymal stem cells (MSC). Gardin et al. explored the possibility that exosomes secreted by hMSCs grown on the different nanotubular Ti surfaces (25, 80 or 140 nm in diameter) could act as biomimetic tools to modulate the biological properties of human umbilical vein endothelial cells (HUVECs) in vitro [3]. The results showed that hMSCs significantly expressed angiogenic-related factors after 7 days of culture on nanotubular Ti compared to untreated Ti substrates and that the nanostructured surface was instrumental in enhancing the release of hMSCs exosomes expressing CD63 and CD81 markers. The latter were efficiently internalized by HUVECs, promoting their migration and differentiation, suggesting that preconditioning hMSCs on nanotubular Ti could be an effective approach to stimulate exosomes secretion and thus ultimately also support faster scaffold integration in vivo.

Nanoscale surface modifications may also have an impact on peri-implant cell fate and implant loading, both of which have an impact on early bone healing. This issue was addressed by De Barros E Lima Bueno et al., who studied how mechanical loading affects healing around implants with nanotopography [4]. At 7 days after implantation in rat tibiae, histomorphometric and gene expression evaluations revealed that implants with a nanostructured surface could achieve a high level of bone formation even under micromotion and limit the inflammatory response at the implant surface compared to standard implants. 

In addition to nanotopography, the composition of the composition certainly plays a key role in determining the final performance of biomedical devices. Poly(3,4-ethylenedioxythiophene):polystyrene sulfonate (PEDOT:PSS) is a widely used conductive polymers for a plethora of applications, including electroactive scaffolds for bone tissue regeneration. As a viable alternative, PEDOT:Nafion is emerging due to its better electrochemical properties than PEDOT:PSS. However, its biocompatibility has not yet been studied. Guzzo et al. investigated the in vitro cytotoxicity of nanostructured PEDOT:Nafion coatings obtained from a water-based formulation of PEDOT:Nafion, using a primary cell culture of rat fibroblasts [5]. The results showed that nanostructured PEDOT:Nafion coatings were not cytotoxic, making the latter a reliable alternative to PEDOT:PSS dispersion, especially in view of long-term in vivo applications.

Magnetic iron oxide nanoparticles are widely used in applications for targeted anti-cancer radiotherapy and imaging techniques using radioisotopes emitting β+, β−, α, and γ radiation. In this context, Nieciecka et al. demonstrated that the conjugation of iron oxide nanoparticles with terbium ions and guanosine-5′-monophosphate (as a thiopurine analogue) were more promising for hypothermia-based treatments than standard unconjugated nanoparticles [6].

Up-conversion rare-earth nanoparticles (UCNPs) are an extremely interesting class of materials due to their high fluorescence intensity as well as the possibility to absorb near-infrared radiation and converting into visible light through non-linear optical processes. Zhang et al. reported a new method for synthesizing Ag-NaYF_4_:Yb^3+^/Er^3+^ UCNPs coated with SiO_2_ using the hydrothermal method [7]. These novel nanoparticles couple both improved light intensity due to the Ag doping and low cytotoxicity due to silica coating, and thus represent a significant advancement in the photothermal therapy and biomedical imaging fields. 

A new and promising strategy for cancer therapy is sonodynamic therapy (SDT). In SDT, effective sonosensitizers are of paramount importance. Ma et al. showed that urchin-shaped copper-based metalloporphyrin liposome nanosystem can be considered highly effective sonosensitizer, as it was found to massively generate reactive oxygen species capable to kill 4T1 tumor cells under ultrasound irradiation [8]. In the field of neuroscience, neuromorphic chips are electronic devices capable of mimicking various brain activities and synaptic functions and are therefore intended to be the building blocks of artificial neural networks. In their work, Hojeong et al. propose biomimetic nanosystems showing different types of resistive switching behaviors, ensuring high pattern-recognition accuracy and short-term plasticity-like behavior [9]. 

This Special Issue also contains four review articles on key topics in the field, which can be very interesting and useful, especially for young researchers involved in this multidisciplinary and highly fascinating field of research. The first review by Duta et al. covers the production of biphasic calcium phosphate materials derived from fish wastes (i.e., heads, bones, skins, and viscera), known as fish discards, in the context of material recycling and the circular economy [10]. This work discusses their promising potential for various applications in the biomedical field, especially orthopedics, where there is development of innovative solutions based on alternative materials of biogenic origin [11,12]. A second review article by Keshvardoostchokami et al. examines the most recent advances concerning the fabrication and application of electrospun nanofibrous polymeric scaffolds [13]. Thanks to the possibility to recapitulate the basic characteristics of the extracellular matrix, natural-, synthetic- and composite-based electrospun scaffolds represent excellent tools for tissue engineering. In particular, their inherent porosity and high surface-to-volume ratio have proven to be highly advantageous for promoting cell adhesion, proliferation and differentiation. Furthermore, thanks to the possibility of finely tuning the orientation and degree of orientation of the nanofibers, the mechanical properties of the scaffolds can be finely tuned and adapted to the desired application. The review by Gherasim et al. considers the applicability of silver nanoparticles, which have been massively used in pharmaceutical and cosmetic industries, anti-infective therapy and wound care, in the food and the textile industries, as therapeutics agents in augmented and alternative strategies for cancer therapy, sensing and diagnosis platforms and restorative and regenerative biomaterials [14]. Finally, the review by Tran et al. focuses on current nanomaterial-based approaches for the treatment of bacterial biofilms formed on the surface of biomedical device [15]. In particular, the latest advances in extracellular polymeric substance disruption and biofilm bacteria killing are discussed to provide guidance for designing next generation antibacterial nano-biomaterials. 

In summary, this Special Issue of *Nanomaterials* covers a wide range of different nanomaterials, including nanostructured coatings, nanoparticles, nanosystems, biomimetic devices and drug carriers that are expected to be the building blocks of next-generation of biomaterials. Although not fully representative of the huge number of different nanomaterials and solutions available for biomedical applications, this Special Issue manages to provide a quick overview of some of the most promising solutions in this rapidly evolving and interdisciplinary field.

## Data Availability

Not applicable.
